# A novel heterozygous germline deletion in MSH2 gene in a five generation Chinese family with Lynch syndrome

**DOI:** 10.18632/oncotarget.19234

**Published:** 2017-07-14

**Authors:** Bin Wu, Wuyang Ji, Shengran Liang, Chao Ling, Yan You, Lai Xu, Min-Er Zhong, Yi Xiao, Hui-Zhong Qiu, Jun-Yang Lu, Santasree Banerjee

**Affiliations:** ^1^ Department of General Surgery, Peking Union Medical College Hospital, Chinese Academy of Medical Sciences and Peking Union Medical College, Beijing 100730, China; ^2^ School of Life Science and Biopharmaceutical, Guangdong Pharmaceutical University, Guangzhou 510006, China; ^3^ Laboratory of Clinical Genetic, Peking Union Medical College Hospital, Chinese Academy of Medical Sciences and Peking Union Medical College, Beijing 100730, China; ^4^ Department of Pathology, Peking Union Medical College Hospital, Chinese Academy of Medical Sciences and Peking Union Medical College, Beijing 100730, China; ^5^ Department of Cell Biology and Medical Genetics, School of Medicine, Zhejiang University, Hangzhou 310000, China

**Keywords:** Lynch syndrome, MSH2 gene, targeted next generation sequencing, novel mutation, DNA mismatch repair gene

## Abstract

Lynch syndrome (LS) is one of the most common familial forms of colorectal cancer predisposing syndrome with an autosomal dominant mode of inheritance. LS is caused by the germline mutations in DNA mismatch repair (MMR) genes including *MSH2, MLH1, MSH6* and *PMS2*. Clinically, LS is characterized by high incidence of early-onset colorectal cancer as well as endometrial, small intestinal and urinary tract cancers, usually occur in the third to fourth decade of the life. Here we describe a five generation Chinese family with LS clinically diagnosed according to the Amsterdam II criteria. Immuno-histochemical staining of MSH2 and MSH6 shows only foci nuclear positive on the surface of the tumor with strong expression of MLH1 and PMS2 with diffuse immunoreactivity. In order to dig into the molecular basis of this LS pedigree, we collected the proband's blood sample, extracted the genomic DNA and applied the genetic screening. As a result, we identified a novel heterozygous deletion in *MSH2* gene by targeted next generation sequencing, which is also proved to be co-segregated among other affected family members by following validation. To our knowledge, this novel heterozygous deletion (c.1676_1679 delTAAA) in *MSH2* gene causes frameshift mutation (p.Asn560Lysfs*29) and leads to the formation of a truncated MSH2 protein which is confirmed to be a deleterious mutation according to the variant interpretation guidelines of American College of Medical Genetics and Genomics (ACMG). Identification of novel DNA mismatch repair (MMR) gene mutations can definitely benefit to the clinical diagnosis and management.

## INTRODUCTION

LS is the most common inherited colorectal cancer (CRC) syndrome accounting for 1% to 13% of all colorectal cancer [1–3] which is characterized by an early onset CRC, with presence of extra-colonic manifestations like endometrial, pancreatic or gastrointestinal cancers [4]. LS is clinically diagnosed on the basis of Amsterdam II criteria, mainly according to the family history [5], but radically associated with germline mutations in DNA mismatch repair (MMR) genes [6, 7] which identified and corrected the DNA base pair mismatches, small deletions and insertions during replication to maintain the genomic stability [8, 9].

Among MMR genes, *MSH2* was the first identified LS-related gene, together with *MLH1*, those two gene mutations lead to majority of LS (> 90% cases) [10–14]. In order to repair the mismatch bases during replication, the *MSH2* interact with *MSH6* or *MSH3* to form the MutSα/β complexes and translocate into nucleus from the cytoplasm through NLS-importin α/β shuttling mechanism [15, 16], then bind to DNA and initiate the repair process. Germline mutations in the MMR genes lead to defective MMR function and results in high rate of spontaneous somatic mutation mostly in microsatellite sequences reflected as microsatellite instability (MSI) [17]. In the international LS database, 500 germline mutations of MMR genes have been enlisted and majority of them are as follows; *MLH1* (50%), *MSH2* (39%) and *MSH6* (7%) [18].

In our case, aiming to understand the molecular basis of this LS pedigree, we undertook a genetic screening for the proband with a panel of 14 genes (*APC, MLH1, MSH2, MSH6, PMS2, AXIN2, BMPR1A, EPCAM, MLH3, MUTYH, PMS1, PTEN, SMAD4, STK11*) associated with Lynch syndrome or colorectal cancer by target exome capture based next-generation sequencing and confirmatory Sanger sequencing, by which we identified a novel heterozygous germline deletion (c.1676_1679 delTAAA, p.Asn560Lysfs*29) in *MSH2* gene, and co-segregating with LS phenotype among all the LS patients in this five generation Chinese family, with autosomal dominant mode of inheritance.

## RESULTS

### Family recruitment and clinical examination

**Figure 1:**
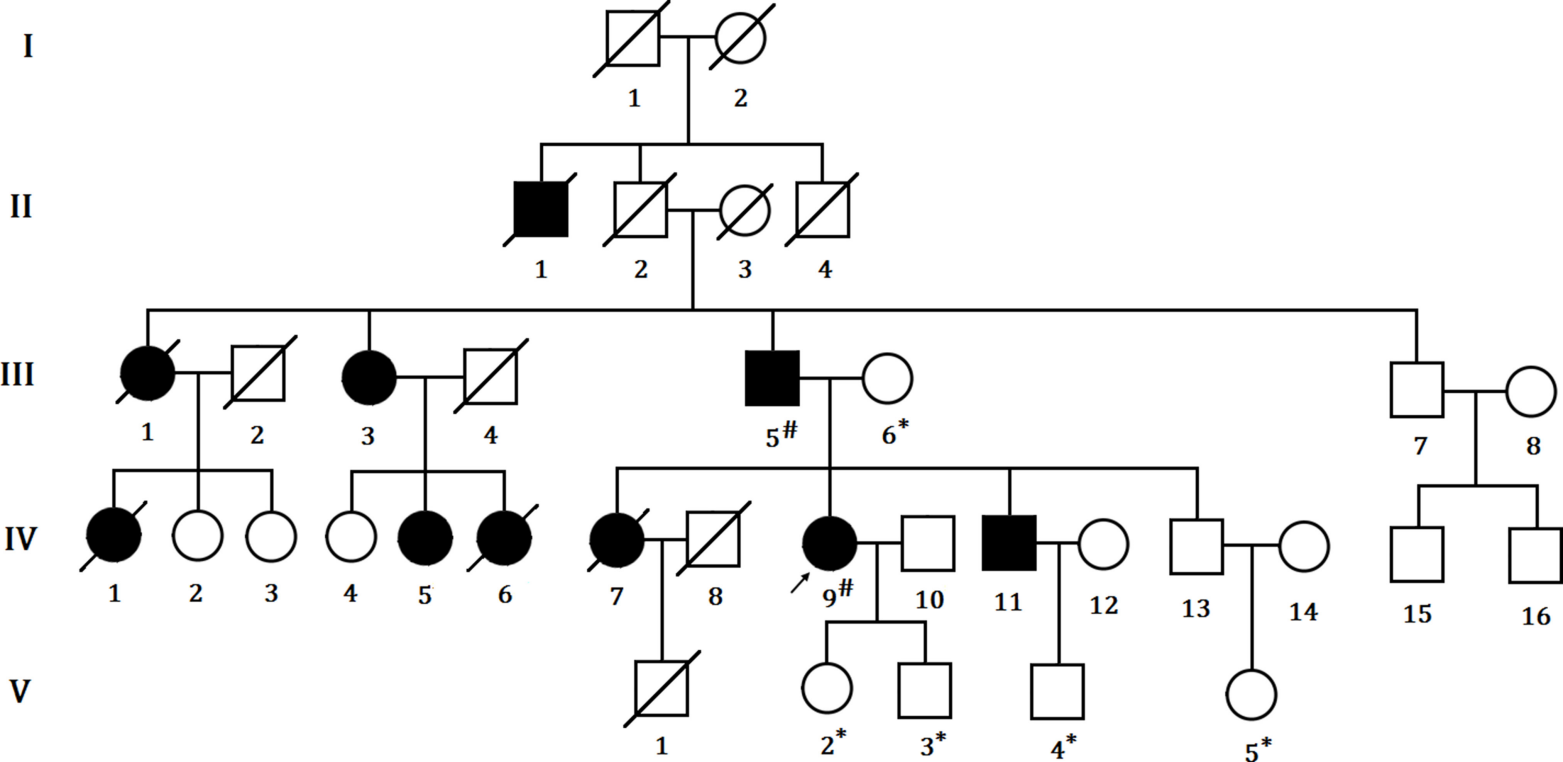
Pedigree structure of the Chinese family with LS The affected family members are indicated with Shading. Squares and circles denoted males and females respectively. Roman numerals indicate generations. Arrow indicates the proband (IV-9). Sign “#” indicates which family members were tested for mutations and found to carry the mutation in the pedigree; Sign “*” indicates which family members were tested and found not to carry the mutation.

**Figure 2:**
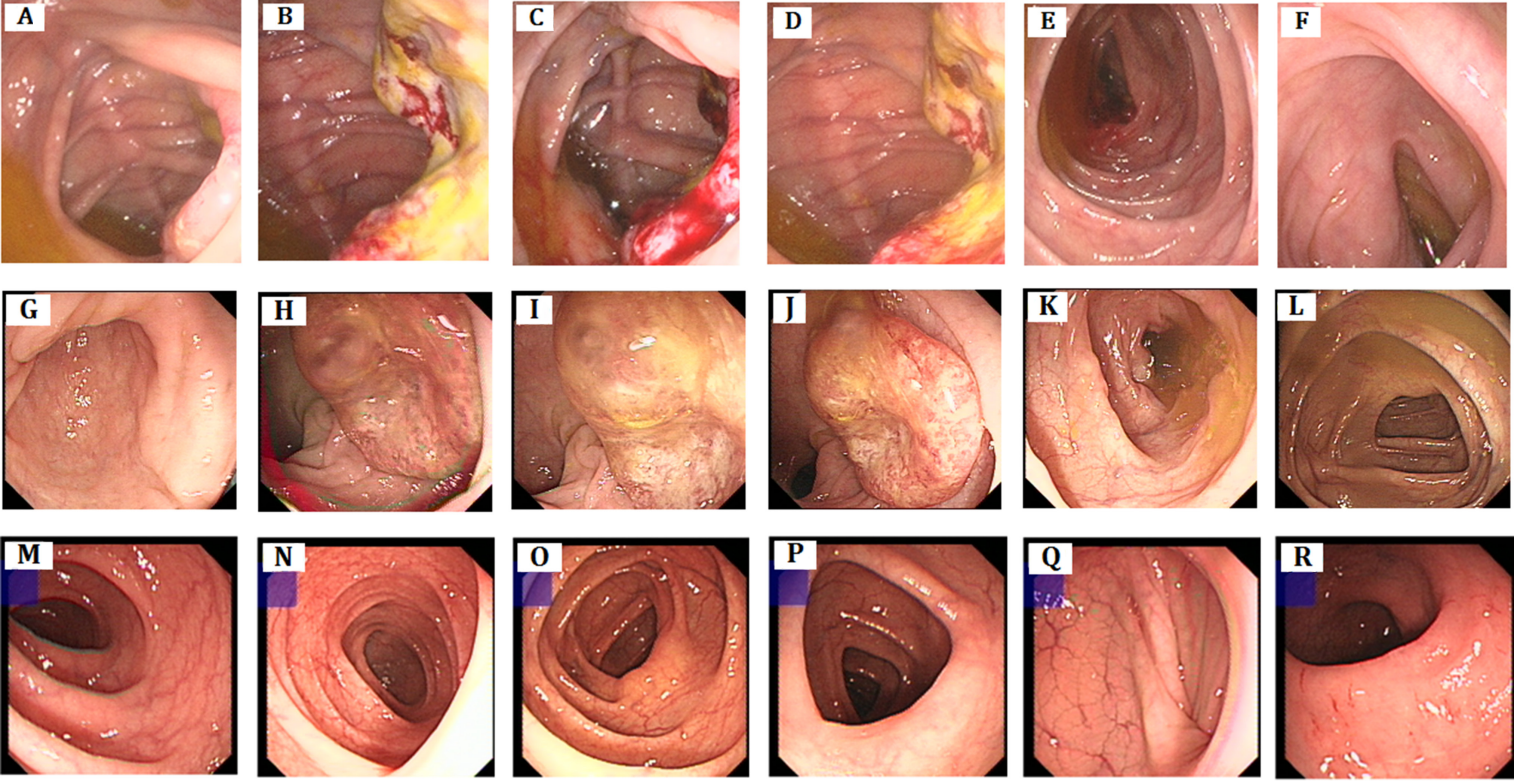
Clinical description. Colonoscopy (**A**–**F**) colon cancer, polypus of colon, There are 3*4 cm of neoplasm and fester of surface adjacent to ileocecal valve, 0.4 cm polypus adjacent to ileocecal (IV-7). (**G**–**L**) rectum cancer, There are about 3.0 cm elevated mucosa at rectum, irregularity, smooth surface, hard texture (IV-9). (**M**–**R**): no abnormality seen (IV-13).

**Figure 3:**
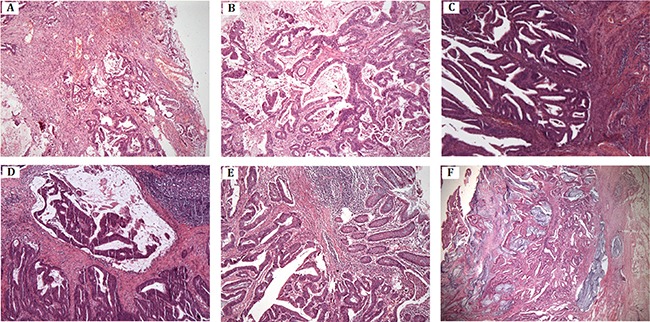
Histopathology and immunostaining (**A**–**B**) Moderately differentiated tubular-papillary adenocarcinoma of right half colon with mucinous areas (< 50%). The tumor penetrates the serosa (IV-7). (**C**) Well-differentiated endometrioid carcinoma of uterine corpus, invading less than half of myometrium (IV-9). (**D**) Moderately differentiated adenocarcinoma of rectum with mucinous component. The tumor invades muscularis propria (IV-9). (**E**–**F**) Moderately differentiated adenocarcinoma of colon with extracellular mucin pools. The tumor extends through the muscularis propria (IV-11).

We identified a five generation Chinese pedigree with 20 members, three of them (III-5, IV-5 and IV-11) were affected with colon cancer, one (III-3) with endometrial cancer and the proband (IV-9) is also with endometrial cancer and rectal cancer (Figure 1). Another 4 affected family members (II-1, III-1, IV-1 and IV-7) had already died from CRC, one affected member (IV-6) died from endometrial cancer. In Table 1, we described the detailed clinical information for all the affected and unaffected members in this family. A comprehensive and comparative colonoscopy results for the affected family members (IV-7 and IV-9) along with an unaffected member (IV-13) are shown in Figure 2, histopathology and immunostaining pictures were shown in Figure 3.

**Table 1:** Clinical characteristics of all the affected and unaffected family members found in our study

Family ID	Sex	WT/MT	Present Age (Years)	Mean age of Diagnosis of LS (Years)	Extra-colonic Features (Diagnosed Years)
**I-1**	M				
**I-2**	F				
**II-1**	M		Died (63)		
**II-2**	M		Died (60)	Undiagnosed	
**II-3**	F		Died (83)		
**II-4**	M		Died (45)	Undiagnosed	
**III-1**	F		Died (69)	68	
**III-2**	M		Died (60)		
**III-3**	F		82		Endometrial cancer (50)
**III-4**	M		Died (70)		
**III-5**	M	MT	79	55	
**III-6**	F	WT	79		
**III-7**	M		74		
**III-8**	F		74		
**IV-1**	F		Died (58)		
**IV-2**	F		55		
**IV-3**	F		50		
**IV-4**	F		57		
**IV-5**	F		56	54	
**IV-6**	F		Died (21)		Endometrial cancer (21)
**IV-7**	F		Died (54)	54	
**IV-8**	M		Died (53)		
**IV-9**	F	MT	55	54	Endometrial cancer (55)
**IV-10**	M		56		
**IV-11**	M		51	45	
**IV-12**	F		49		
**IV-13**	M		49		
**IV-14**	F		46		
**IV-15**	M		54		
**IV-16**	M		51		
**V-1**	M		Died (28)	Undiagnosed	
**V-2**	F	WT	31		
**V-3**	M	WT	29		
**V-4**	M	WT	26		
**V-5**	F		23		

### Clinical description

Here we described the detailed clinical manifestations of the proband (IV-9), two affected family members (IV-7 and IV-11) and one unaffected (IV-13) family member.

IV-9: The proband is a 54-year old female with a history of blood in the stool for more than 1 month. Before colonoscopy, she had a digital rectal examination (DRE) and there was no stenosis, lumps in rectum, but after the DRE examination, finger has been stained by dark red blood. During the colonoscopy, we found a 3.0 cm hard and irregular mucosa uplift at 10 cm away from the anal margin, however the ileocecal valve, ascending, transverse, descending and sigmoid mucosa were smooth and clear in texture without ulcers and space lesions.

Pathology of colonoscopy biopsy proved to be moderate differentiated adenocarcinoma. After the radical resection of rectal cancer (Dixon), post-operative pathology proved to be well to moderate differentiated adenocarcinoma with mucinous component invaded to muscle layer. Fortunately, the upper and lower ends of the cut edge and circumferential resection margin were clear and the lymph nodes showed chronic inflammation. Immuno-histochemical staining for the proband's tumor cell showed that strong expression of the MLH1 and PMS2 with diffuse immunoreactivity. However, the immunostain of MSH2 and MSH6 showed only foci nuclear positive on the surface of the tumor. Postoperative radiotherapy and chemotherapy was recommended.

One year later, at the age of 55 years, this proband complained with the vaginal irregular bleeding and admitted to hospital again. With the well to moderately differentiated adenocarcinoma pathology result, she received a laparoscopic total hysterectomy (hysterectomy + pelvic lymph node dissection + abdominal aortic lymph node dissection). Post operation pathology presented endometrial well-differentiated endometrioid carcinoma, invaded to superficial muscle (through muscle wall) without involvement of the lower uterine segment and bilateral uterine cervix, bilateral fallopian tube and ovary tissue showed no lymph node metastasis. 6 routine of concurrent chemoradiotherapy (paclitaxel + carboplatin) were performed after surgery.

IV-7: IV-7 is a 54-year-old female with a history of abdominal mass for more than 6 months and identified a 3 ×4 cm ulcerative neoplasm located at the lip-shaped ileocecal valve by colonoscopy, which maintain, normal relaxation and constriction function, furthermore, a 0.4cm polyp nearby the appendix fossa is visible. No colonoscopy biopsy was performed. Then, she underwent a hemicolectomy surgery with the post-operative pathology which showed a 4 × 4 cm tumor as moderately differentiated tubular-papillary adenocarcinoma with mucinous areas with neuroendocrine differentiation (< 50%). The tumor penetrates the serosa with vascular invasion, without nerve invasion. Tumor cells were visible at the peripheral margin of the operated tissue.

IV-11: IV-11 is a 45-year old male admitted to the hospital with abdominal pain and melena for 3 months. Colonoscopy showed a 5 cm long semi-circular cauliflower-like mass located at the transverse colon near the spleen area, protruding to the cavity with congestion, erosion and ulcers. After the transverse colon cancer resection, the pathology showed moderately differentiated adenocarcinoma, mucinous adenocarcinoma (4.5 cm) invaded muscle layer, without peri-colon lymph node metastasis. A 12-routine chemotherapy has been undertaken after surgery.

IV-13: IV-3 is a 49-year old male without any clinical symptoms. In order to ensure the current health status, he received a colonoscopy screening and showed no abnormalities for now.

### Immunohistochemistry analysis

**Figure 4:**
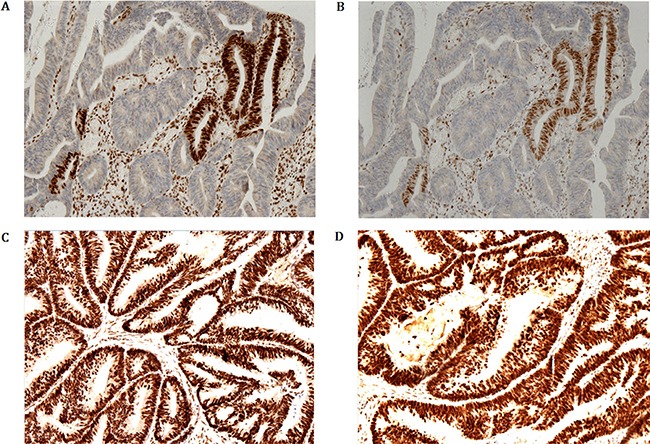
Immunohistochemical staining demonstrating MLH1 and PMS2 expression of tumor cells is strong and diffuse immune-reactivity However, the immunostaining of MSH2 and MSH6 shows only foci nuclear positive on the surface of the tumor in proband (IV-9).

Immuno-histochemical staining demonstrating MLH1 (Figure 4C) and PMS2 (Figure 4D) expression of tumor cells is strong and diffuse immune-reactivity. However, the immune-stain of MSH2 (Figure 4A) and MSH6 (Figure 4B) shows only foci nuclear positive on the surface of the tumor in proband (IV-9).

### Identification and characterization of candidate mutation

A heterozygous novel deletion; c.1676_1679 delTAAA, p.Asn560Lysfs*29 in *MSH2* gene [NCBI Reference sequence NM_000251] was identified in proband (IV-9) by targeted next generation sequencing. This novel heterozygous deletion is co-segregated with the LS phenotypes in the proband (IV-9) and amongst the affected family (III-5) members, but absent in the unaffected family members (III-6, V2, V3, V4, V5). We did not identify this mutation in the 100 normal control of the same ethnic origin, gender and age range.

### Confirmation of the novel deletion by sanger Sequence

**Figure 5:**
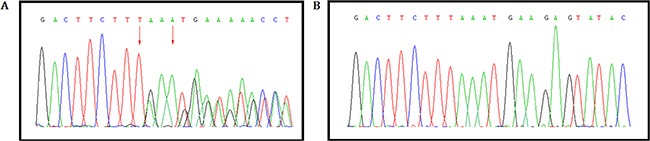
Validation of the novel heterozygous germline deletion in all the affected by Sanger sequence (**A**) A heterozygous novel deletion; c.1676_1679 delTAAA, p.Asn560Lysfs*29 in *MSH2* gene [NCBI Reference sequence NM_000251] was identified in proband (IV-9) and amongst the affected family (III-5) members, (**B**) But absent in the unaffected family members (III-6, V2, V3, V4, V5).

This novel heterozygous deletion; c.1676_1679 delTAAA, p.Asn560Lysfs*29 in *MSH2* gene was confirmed by Sanger sequencing (Figure 5).

## DISCUSSION

**Figure 6:**
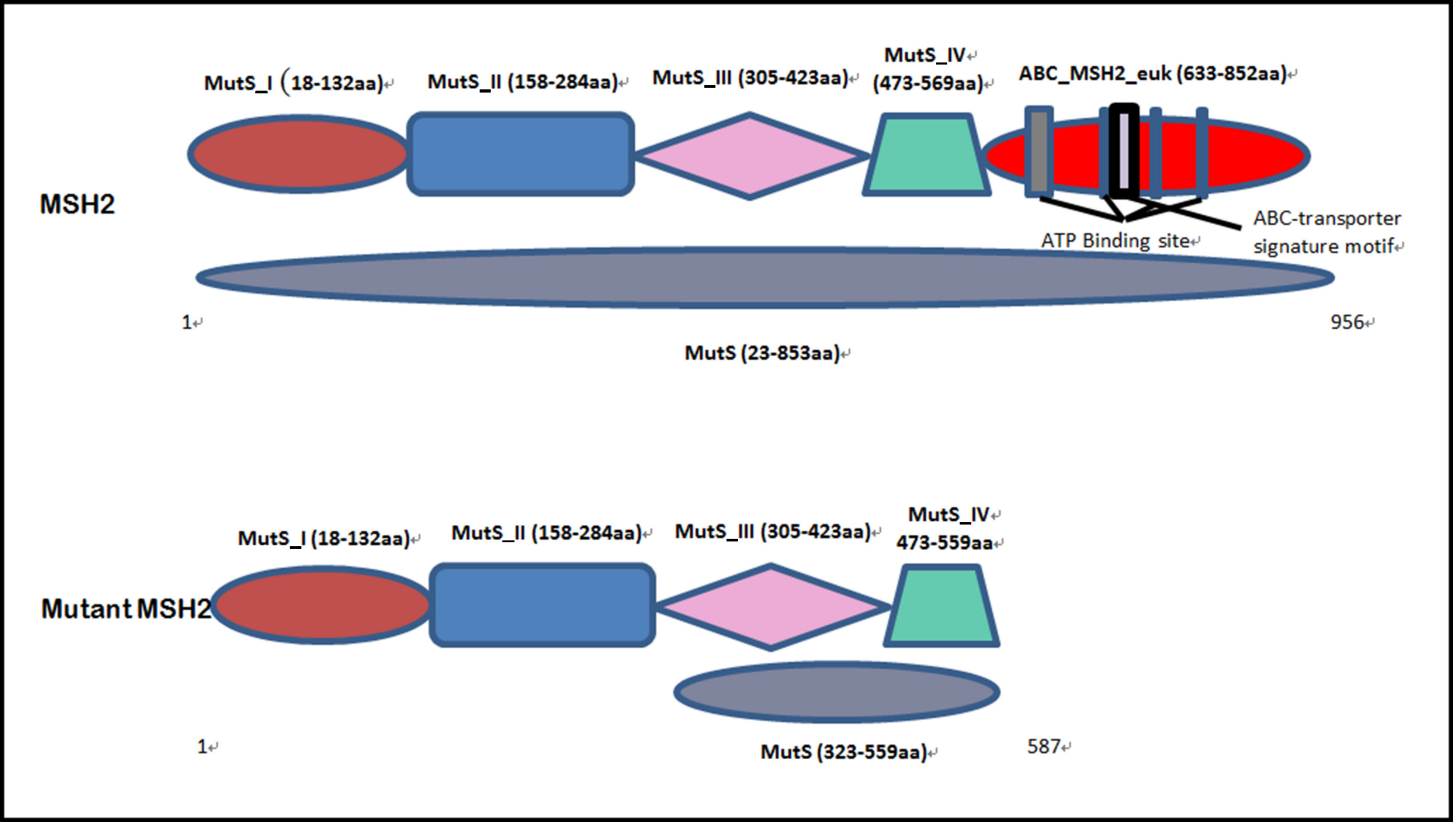
Schematic presentation of both wild type and mutated MSH2 protein domain

In our study, we found a novel heterozygous deletion (c.1676_1679 delTAAA; p.Asn560Lysfs*29) [NCBI Reference sequence NM_000251] of the human *MSH2* gene in the proband (IV-9) and among the affected family members [III-5] in a five generation Chinese family with LS. This heterozygous novel deletion of MSH2 gene has not presented in the ExAC database. This deletion mutation results in the formation of truncated *MSH2* protein by the presence of a premature termination codon. The wild type and mutant MSH2 protein is schematically presented in Figure 6.

### Function of MMR genes

Fishel et al. and Leach et al. first independently reported the mutation of MMR genes is associated with Lynch syndrome according to their study that showed the mutation of MSH2 gene causes Lynch syndrome [20, 21].

In addition, MMR genes are associated with several cellular functions such as repair the DNA mismatch error, DNA double strand breaks, DNA destabilization and apoptosis. Therefore, the MMR proteins are very significant in maintain both DNA stability and cell-cycle regulation. Hence, mutations of MMR genes lead to loss or defected MMR protein further results in decreased apoptosis as well as increased cell survival, activated the potential increase in damage-induced mutagenesis. Finally, damage-induced mutagenesis leads to a selective growth advantage to the cell, followed by an increased susceptibility to tissue-specific cancers.

### Screening of MMR genes

However, the main function of MMR gene is to repair the mismatched bases in DNA during DNA replication to maintain the genetic stability. Non-functional MMR protein causes instability of microsatellite, the repetitive DNA sequences, resulted phenotype is called microsatellite instability (MSI). Among LS patients, the microsatellite instability is tested in the tumor, harboring pathogenic mutations of MMR gene.

According to the Bethesda guidelines, a panel of five microsatellites was recommended as a reference panel for screening of MMR genes caused MSI [22]. In addition, based on MSI result, there are three levels of microsatellite instability: MSI-High (MSI-H), with > 30% of tested loci are unstable, MSI-Low (MSI-L), with < 30% of tested loci are unstable; and microsatellite stability (MSS), when no tested loci are unstable. For most instances, MSI-L and MSS tumors are lack of familial MMR gene mutations. These guidelines were revised in 2002 to clarify a number of issues concerning the original Bethesda criteria and to further aid in the identification of families for additional testing [23].

Moreover, apart from MSI, there is another technology; called immunohistochemistry (IHC) which can detect the expression of the MMR proteins in tumor tissues. However, for individuals with tumor, IHC is the first line of histopathological test recommended for clinical diagnosis. According to the result of IHC, genetic screening should be recommended to patients who absence or express low level of MMR protein in order to discover the pathogenic germline alteration underlying the disease phenotype. In addition, for detection of MMR protein deficiency, IHC is a more specific and sensitive than MSI testing, as IHC is a faster and cost-effective test which can directly detect the mutated gene.

### Immunohistochemistry (IHC) of tumor tissue

The expression or the presence of MMR protein in tumor tissues is identified by IHC. In functional mode, the MMR proteins bind together to form the dimer; MSH2 protein interact with MSH6 protein to form a hetero-dimer, while MLH1 protein form the dimer with PMS2 protein. Furthermore, MSH6 and PMS2 proteins are unstable till they form the dimer with the MSH2 and MLH1 protein respectively. As the result, germline mutations of *MSH2* or *MLH1* gene lead to the formation of defective MSH2 or MLH1 proteins unable to further interact with MSH6 and PMS2 results in loss of function of the proteins MSH2/MSH6 and MLH1/PMS2 respectively. On the other hand, germline mutations in MSH6 and PMS2 won't result in loss of MSH2 or MLH1 protein expression as MSH2 and MLH1 proteins are more stable as monomer.

### *MSH2* gene mutation

The germline mutations of MSH2 associated with LS, are mostly results into formation of truncated MSH2 protein but single amino acid substitution (20–25%) is also possible [18, 19]. A large number of pathogenic *MSH2* gene mutations have been reported to be associated with LS in different countries and ethnic groups (http://chromium.lovd.nl/LOVD2/colon_cancer/home.php?select_db=MSH2).

Till date, 5327 sequence variants of the *MSH2* gene have been reported in the World population. Among these, 1018 are unique variants which were identified out of 5104 individuals. Among 5327 sequence variants, of the *MSH2* gene, the majority is substitutions (Total:1804; confirmed: 787, predicted: 1017), but frameshift (Total: 1001; confirmed: 356, predicted: 645), nonsense (Total: 594l; confirmed: 248, predicted: 346), deletion (Total: 421; confirmed: 352, predicted: 69) are also reported

Until now, 119 sequence variants of the *MSH2* gene have been reported in the Chinese population. Among these, 77 unique variants were identified out of 116 individuals. Among the 119 sequence variants of the *MSH2* gene, the majority is substitutions (51), but frameshift (25), nonsense (10), deletion (27), insertion (9) and duplication (4) are also reported (http://www.genomed.org/lovd2/variants_statistics.php).

### Genotype–phenotype correlation

Genotype–phenotype correlation studies are very significant and enable us to define the most likely phenotype associated with a given mutation. The identification and characterization of *MSH2* mutation carriers with a well diagnosed phenotype will allow us to establish specific surveillance programs and prophylactic treatment.

Lynch syndrome usually manifests with a spectrum of clinical symptoms with diversity frequencies of according to different MMR gene mutations. Recently, some reports have suggested the families with MSH2 mutations are more prone to develop extracolonic cancers compared to those with MLH1 mutations, whereas cases associated with MSH6 mutations have the highest risk of developing endometrial cancer [33]. Furthermore, ethnic-specific differences in phenotypes are also classified in Lynch syndrome. Endometrial cancer and stomach cancer is predominant as extracolonic manifestations in patients with LS in western countries and in Southeast Asia [24, 25]. The frequencies of MMR gene mutations are also sometimes correlated with different population [26]. These specific characteristics identified in the patients with Lynch syndrome are very significant for clinical or molecular diagnosis and follow-up visit of those affected patients as well as unaffected mutation carriers. The comprehensive genotype-phenotype correlation studies allow us to provide more specific treatments for the patients with LS [27].

Moreover, Pérez-Cabornero et al., has reported that different type of the mutation (point or large rearrangements) is not correlated with the occurrence of extracolonic manifestations, age of onset or type of tumor in families with LS [28]. Amongst LS patients, carriers of MLH1 mutations showed high frequency of CRC while carriers of MSH2 mutations showed high frequency of extra-colonic cancer. Carriers of MSH2 mutations also presented with high frequency in developing multiple tumors.

As for different MMR gene mutations, germline mutations of *MLH1* and MSH2 accounting for almost 50% and 40% cases of Lynch syndrome respectively [29, 30]. Meanwhile, germline mutations of *MSH6* and PMS2 accounts for 7–10% and less than 5% cases of Lynch syndrome respectively [31, 32].

In conclusion, the present study describes a heterozygous novel deletion mutation in *MSH2* gene in a five generation Chinese family with LS. Our study expands the spectrum of the germline mutations of *MSH2* gene in the Chinese population. This novel finding contributes to a more comprehensive database of germline mutations in *MSH2* gene that could be used in further molecular diagnosis, risk assessment, susceptible treatment for LS patients.

## MATERIALS AND METHODS

### Ethical statement

Family members of this five generation Chinese family have given written informed consent as they are participating in this study. The Ethical Committee of the Peking Union Medical College Hospital, China, reviewed and approved our study protocol in compliance with the Helsinki declaration. Diagnosis of the patients for Lynch syndrome was made by oncologists, on the basis of Amsterdam II criteria, clinical test reports and detailed family pedigree.

### Patients and pedigree

A five generation Chinese family with Lynch syndrome (Figure 1), diagnosed and treated in the Department of General Surgery, Peking Union Medical College Hospital, China, were enrolled in our study. Clinical diagnosis of Lynch syndrome was established in this family by endoscopic screening after the proband (IV-9) presented to Peking Union Medical College Hospital, China with LS. The diagnostic standard or criteria for patients with Lynch syndrome was as follows: Diagnosis of Lynch syndrome is based on the Amsterdam criteria II (discussed in detail in the “Introduction” part).

### Targeted exome-based next-generation sequencing and variant identification

DNA samples obtained from the proband (IV-9) were sequenced using target exome-based next-generation sequencing. Roche NimbleGen's (Madison, USA) custom Sequence Capture Human Array was used to designed to capture 98480 kb of targeted sequence, covering 181 exons and flanking sequence (including the 100 bp of introns) of 14 genes (*APC, MLH1, MSH2, MSH6, PMS2, AXIN2, BMPR1A, EPCAM, MLH3, MUTYH, PMS1, PTEN, SMAD4, STK11*) which is associated with colorectal cancer (CRC) and yielded an average of 6366534 reads per sample, with approximately 68.78% mapping to the targeted regions. The average sequencing depth of the target area is 464.68 with 99.46% coverage. The procedure for preparation of libraries was consistent with standard operating protocols published previously. In each pooling batch, 10 to 33 samples were sequenced simultaneously on Illumina HiSeq 2500 Analyzers (Illumina, San Diego, USA) for 90 cycles (specially designed by us for this study). Image analysis, error estimation, and base calling were performed using Illumina Pipeline software (version 1.3.4) to generate raw data. The raw reads were screened to generate – clean reads‖ followed by established filtering criteria. Clean reads with a length of 90 bp were aligned to the reference human genome from the NCBI database (Build 37) using the Burrows Wheeler Aligner (BWA) Multi-Vision software package with output files in - bam‖ format. The bamdata were used for reads coverage in the target region and sequencing depth computation, SNP and INDEL calling, and CNV detection. First, a novel three-step computational frame work for CNV was applied. Then, SNPs and INDELs were called using SOAPsnp software and Sam tools pileup software, respectively. A SNP or INDEL would be filtered out if it could not follow the criterion: supported by at least 10 reads and > 20% of the total reads. The frequency filter was set at 0.05. If a SNP frequency was more than 0.05 in any of the four databases (dbSNP, Hapmap, 1000 Genomes Project, the 124 healthy reference samples sequenced in this study), it would be regarded as a polymorphism, but not a causative mutation.

Last, SNVs were retrieved in The Human Gene Mutation Database (http://www.hgmd.cf.ac.uk/ac/index.php) and the Leiden Open Variation Database (http://www.lovd.nl/3.0/home), and then labeled as reported or novel.

### Sanger Sequence

To validate the true positive of the mutation, Sanger sequencing was performed. Primers flanking the candidate loci were designed based on the reference genomic sequences of Human Genome from GenBank in NCBI and synthesized by Invitrogen, Shanghai, China. PCR amplification was carried out in ABI 9700 Thermal Cycler. PCR products were directly sequenced on ABI PRISM 3730 automated sequencer (Applied Biosystems, Foster City, CA, USA). Sequence data comparisons and analysis were performed by DNASTAR SeqMan (DNASTAR, Madison, Wisconsin, USA).

The novel heterozygous deletion identified in *MSH2* gene through targeted next generation sequencing were verified through Sanger sequencing using the primers: F-5′- AAGGAGTTGTTCGTTTTCCACTT -3′, R-5′- TTACCAAAAGCCAGGTGACATTC -3′. The reference sequence NM_000251 of *MSH2* was used.

### Mismatch repair protein immunohistochemistry

Immunoperoxidase staining was performed on formalin-fixed tissue. Immunohistochemistry with MMR protein has performed by using the standard streptavidin–biotin–peroxidase procedure. Primary monoclonal antibodies against *MLH1* (clone G168-728; BD PharMingen, San Diego, CA, USA; 1:200), *MSH2* (clone FE11; Oncogene Research Products, Cambridge, MA, USA; 1:100), *MSH6* (clone 44; BD Transduction, San Jose, CA, USA; 1:200), and *PMS2* (clone A16-4, BD Biosciences, San Jose, CA, USA; 1:10) were applied to 5-μm-thick formalin-fixed, paraffin-embedded whole tumor sections.
